# Deep learning-based prediction of deliverable adaptive plans for MR-guided adaptive radiotherapy: A feasibility study

**DOI:** 10.3389/fonc.2023.939951

**Published:** 2023-01-18

**Authors:** Laura Buchanan, Saleh Hamdan, Ying Zhang, Xinfeng Chen, X. Allen Li

**Affiliations:** Department of Radiation Oncology, Medical College of Wisconsin, Milwaukee, WI, United States

**Keywords:** adaptive radiation therapy, MR-guided adaptive radiation therapy, online replanning, real-time adaptation, deep-learning

## Abstract

**Purpose:**

Fast and automated plan generation is desirable in radiation therapy (RT), in particular, for MR-guided online adaptive RT (MRgOART) or real-time (intrafractional) adaptive RT (MRgRART), to reduce replanning time. The purpose of this study is to investigate the feasibility of using deep learning to quickly predict deliverable adaptive plans based on a target dose distribution for MRgOART/MRgRART.

**Methods:**

A conditional generative adversarial network (cGAN) was trained to predict the MLC leaf sequence corresponding to a target dose distribution based on reference plan created prior to MRgOART using a 1.5T MR-Linac. The training dataset included 50 ground truth dose distributions and corresponding beam parameters (aperture shapes and weights) created during MRgOART for 10 pancreatic cancer patients (each with five fractions). The model input was the dose distribution from each individual beam and the output was the predicted corresponding field segments with specific shape and weight. Patient-based leave-one-out-cross-validation was employed and for each model trained, four (44 training beams) out of five fractionated plans of the left-out patient were set aside for testing purposes. We deliberately kept a single fractionated plan in the training dataset so that the model could learn to replan the patient based on a prior plan. The model performance was evaluated by calculating the gamma passing rate of the ground truth dose vs. the dose from the predicted adaptive plan and calculating max and mean dose metrics.

**Results:**

The average gamma passing rate (95%, 3mm/3%) among 10 test cases was 88%. In general, we observed 95% of the prescription dose to PTV achieved with an average 7.6% increase of max and mean dose, respectively, to OARs for predicted replans. Complete adaptive plans were predicted in ≤20 s using a GTX 1660TI GPU.

**Conclusion:**

We have proposed and demonstrated a deep learning method to generate adaptive plans automatically and rapidly for MRgOART. With further developments using large datasets and the inclusion of patient contours, the method may be implemented to accelerate MRgOART process or even to facilitate MRgRART.

## 1 Introduction

With the introduction of the MR-Linac into the clinic has come the opportunity for robust MR-guided online adaptive radiation therapy (MRgOART), which is performed by adapting the reference treatment plan created based on the simulation image to the anatomy, e.g., the contours of the lesion and organs at risk (OARs), of the day and delivering the adaptive plan to the patient for the fraction (1, 2). A fast and robust online adaptive replanning process is highly desirable to take full advantage of the MR-Linac capabilities. The process is complex, including daily image acquisition, image segmentation, plan generation, and plan evaluation and verification. With the current technologies, the MRgOART process takes 30-90 minutes (3, 4). A contributing factor to this lengthy process is the plan generation time, which can take up to 20 minutes. Such a labor intensive and time-consuming replanning process has prevented MRgOART from entering routine clinical practice.

During the treatment delivery of MRgOART, the non-ionizing radiation nature of MRI allows for continuous 2D (cine) or periodic 3D imaging, permitting localization of tumor targets and/or OARs (5). It has been well documented that intrafraction anatomical changes exist in various tumor sites and such changes can lead to substantial differences between the intended dose distribution and the delivered dose distribution (6, 7). Particularly in the abdomen, movement of abdominal organs due to digestion can be difficult to predict, and real-time management of these intrafraction variations of the tumor, OARs, and air cavities is crucial in MRgOART to ensure accurate delivery of the intended dose. A desirable approach, termed real-time adaptive radiation therapy (RART), is to adapt the treatment plan in near real-time (at least a few times) based on the recent image during one treatment session. Ultra-fast plan generation is essential to facilitate RART. An approach to reduce plan generation time is to use a library of plans created offline, avoiding the lengthy inverse plan optimization commonly used in conventional treatment planning systems (TPS). In the online process, the most suitable plan is selected from the library for the daily anatomy and then the dose from the atlas patient is mapped to the new patient using a machine learning approach based on radiomic features to estimate the dose in each voxel of the patient (8). Taking it a step further, the target dose distribution derived from the atlas patient is also used in commercial dose mimicking software (RaySearch Laboratories, Stockholm, Sweden) using a collapsed cone convolution dose engine (9).

Spurred by recent advances in computational power and high-level neural networks APIs, deep learning (DL) is rapidly changing the field of radiation oncology by offering big data-driven approaches to solve complex problems including auto segmentation, dose prediction, quality assurance, and automated treatment planning (10–12). Recent progress has been made in predicting a desirable dose distribution for a given patient anatomy using DL-based approaches (13–19). Using the patient contours as input, patient-specific dose distributions can be generated without the need of an inverse plan optimization process. To our knowledge, there has not been effort reported to convert such a desirable dose distribution to a deliverable plan in terms of beam parameters, e.g., beam angles, aperture shapes, and aperture weights [monitor unit (MU) number] using a stand-alone DL model.

Several recent studies have developed DL networks to convert a desirable dose distribution to a fluence map. For example, Wang et. Al (20) used two convolutional neural networks (CNN) to (1) predict a desirable dose distribution based on patient anatomy and (2) translate the predicted dose distribution to a fluence map. Lee et al. (21) trained a deep neural network to directly predict fluence maps based on patient anatomy for intensity modulated radiotherapy (IMRT) prostrate cases. Ma et. Al (22) developed a DL network to predict volumetric arc therapy (VMAT) fluence maps for both head and neck cases as well as prostate cases in less than 1 s. However, in all these studies the fluence map must be transported into a conventional treatment planning system (TPS) to perform multi-leaf collimator (MLC) sequencing and calculate a final dose calculation. This additional step requires extra time and effort on the part of the planner.

Here, we propose a novel, fast plan generation method particularly for online adaptive replanning, where a previous plan based on either simulation (reference) image or the daily image of a previous fraction is available. The method uses a DL network to rapidly predict an MLC leaf sequence based on a desirable dose distribution and previous plan. Our newly proposed method bypasses the fluence map prediction and converts a target dose distribution directly to a deliverable leaf sequence, e.g., field segment shapes and weights (MUs).

We further propose that future clinical implementation of our method will utilize transfer learning. This will allow the model to be continuously updated with the most recent high-quality plans generated based on the recent image sets during the treatment. On the day of treatment, a daily MR image would first be acquired and registered to the recent image to determine if MRgOART is warranted. If the decision to proceed with adaptive planning is made, a dose prediction would be made using methods outlined in several previous works (13–19), which are not within the scope of our present study. The dose prediction serves as the input to our proposed model to predict a deliverable plan which includes aperture shapes and corresponding MU numbers using the deep learning models that are the scope of this paper. Our proposed method avoids the lengthy fluence map and segment shape/weight reoptimization. Using deep learning, we may be able to generate deliverable plans in a few seconds rather than the tens of minutes currently required. Such an ultra-fast generation of deliverable plans may be used for online adaptive replanning to address inter-fractional variations or even real-time adaptation for intra-fractional changes.

This paper demonstrates the feasibility of our proposed method and is organized as follows: Section 2 will describe the proposed methods and the data used, including study design, data collections and pre-processing, the patient cohort used for training and testing as well as the validation method, and the auto-plan generation step. Results obtained will be presented in Section 3, and discussions on how the proposed method may be applied to MRgOART will be provided in Section 4. Section 5 will conclude with the strengths and weaknesses of this study as well as what the next steps will be.

## 2 Materials and methods

### 2.1 Study design

**Figure 1:**
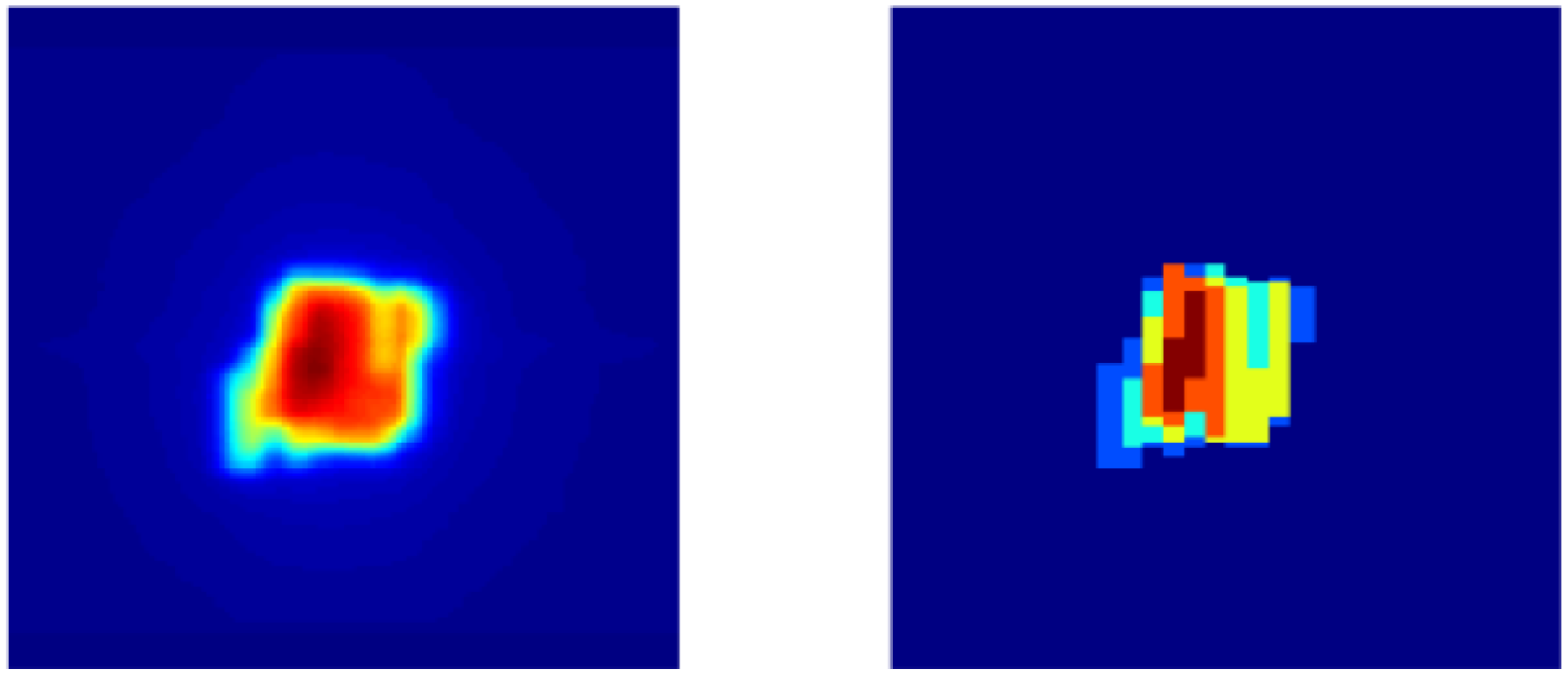
Left: 2D dose map of single beam viewed from beam’s eye view. Right: Sum of individual ground truth field segments projected at isocenter.

Every beam in a step-and-shoot IMRT plan is composed of several individual field segments, and each field segment has a specific shape defined by the MLC openings and MU weight. Field segments can be thought of as a series of simple 2D images. As shown in **Figure 1**, if viewed from the beam’s eye view, the sum of the field segments delivered by the LINAC for a single beam approximately resembles the 2D representation of the dose deposited by that beam. Therefore, we hypothesized that individual field segments could be predicted if given a target dose distribution of a single beam, framing the problem as an image-to-image translation task. In our study, we model the relationship between the total dose deposited by a single beam and the individual field segments that correspond to that beam.

Deep neural networks can easily learn the mapping from one input image to another image as in semantic segmentation (23). Specifically, conditional generative adversarial networks (cGANs) have a robust architecture fit for a variety of image-to-image translation tasks. Our network architecture is adapted from the pix2pix architecture (24), which was found to be effective on a wide range of image translation problems including semantic labels to photos, photos to semantic labels, and map to aerial photos. Mahmood et al. (25) used the pix2pix architecture to predict 3D dose distributions of oropharyngeal cancer patients from patient contours and the planning CT. The adaptability of the cGAN architecture is because the model learns a loss that is specific to each application, without the need for manual tuning typical of stand-alone convolutional neural networks. It was also shown that acceptable results can be obtained using as little as 400 training images.

In general, a GAN is composed of two neural networks: a generator (G) and discriminator (D). Training a cGAN can be thought of as a two-player game, where G tries to generate images that cannot be distinguished from synthetic ones, and D is trained to classify images as either a true sample from the training data or a synthetic image produced by the generator. The cGAN loss is defined as


(1) $$Loss_{cGAN}\left(G,D\right)=\;\sum_{x,y}[\log D\left(x,y\right)]+\sum_{x,y}[\log\left(1-D\left(x,G\left(x\right)\right)\right]\;\;$$

During training, G and D update their weights one at a time in an adversarial manner such that D is trained to maximize the log probability of real images, D(x,y), and the log of the inverse probability of synthetic images, 1 – D(x,G(x)). In other words, the discriminator “wins” the game when it detects the synthetic images 100% of the time. On the other hand, G is trained to minimize the L1 loss between the synthetic images and ground truth images. Training is complete when an equilibrium is reached between the G and D loss. The final objective function, F, is defined as


(2) $$F=arg\;min_{G}max_{D}Loss_{cGAN}\left(G,D\right)+\;\lambda Loss_{L1}\left(G\right)$$

where λ is a hyperparameter that gives weight to the L1 loss of the generator.

The G and D architectures of our model are adapted from Isola et al. For the generator, we used 6 down-sampling blocks that each consist of a convolution (kernel = 4x4, stride = 2x2), batch normalization, and a leaky ReLU activation function. The up-sampling blocks used transposed convolutions (kernel = 4x4, stride = 2x2), batch normalization, and a ReLU activation function. The generator architecture is like UNet (26) with skip connections between the down-sampling and up-sampling blocks to encourage the learning of both high and low-level features. Like the original Isola paper, instead of introducing a random noise vector as additional input data, random dropout was used in the generator to introduce noise and prevent over-fitting of the data.

**Figure 2:**
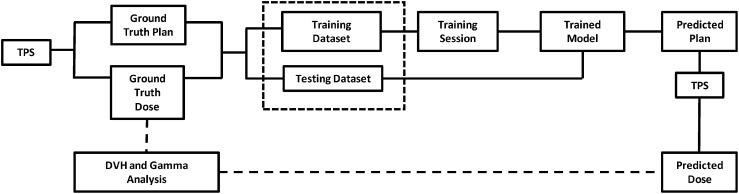
High-level workflow of study design.

The TPS (Monaco, Elekta AB) used a Monte Carlo algorithm to optimize a ground truth plan based on a patient’s anatomy (an image set and contours of lesion and OARs). For our study, the ground truth dose distribution defined the target dose distribution and was used to train the model. However, once the model is trained, any target dose distribution that considers the patient’s anatomy could be used as input to the model. The outputs of the model were the individual field segment shapes with specific MU numbers corresponding to each beam. These data were used to generate new MLC control points to form a newly predicted RTPlan dicom. The predicted plan was then transferred back into the TPS to calculate what the dose would have been if delivered to the test patient. DVH comparison and gamma analysis (3%, 3mm, 5% threshold) between the target dose distribution and recalculated dose distribution from the predicted plan were performed to assess the predicted plan quality. This workflow is summarized in **Figure 2**.

### 2.2 Data collection and preprocessing

A total of 50 daily adaptive step-and-shoot IMRT plans, including dose distributions and aperture shapes and weights, created for 10 pancreatic cancer patients (each with five treatment fractions) during routine MRgOART with an MR-Linac (Unity, Elekta AB), were retrospectively selected as the ground truth data for the DL model training and testing. The prescription dose for eight of the ten patients was 35 Gy delivered in five fractions, with V(3500 cGy) ≥ 95% for PTV coverage of six of these eight, and the remaining two of these eight prescribed 68% and 97.4% PTV coverage. Two of the ten patients were prescribed 33 Gy with V(3300 cGy) ≥ 95% for PTV coverage to be delivered in five fractions. Nine of the 10 cases were pancreatic head tumors and one case was a pancreatic tail tumor. All plans consisted of the same 11 beam angles (5°, 25°, 60°, 90°, 155°, 175°, 195°, 260°, 290°, 310°, 345°), and were optimized using an online replanning TPS (Monaco, Elekta).

**Figure 3:**
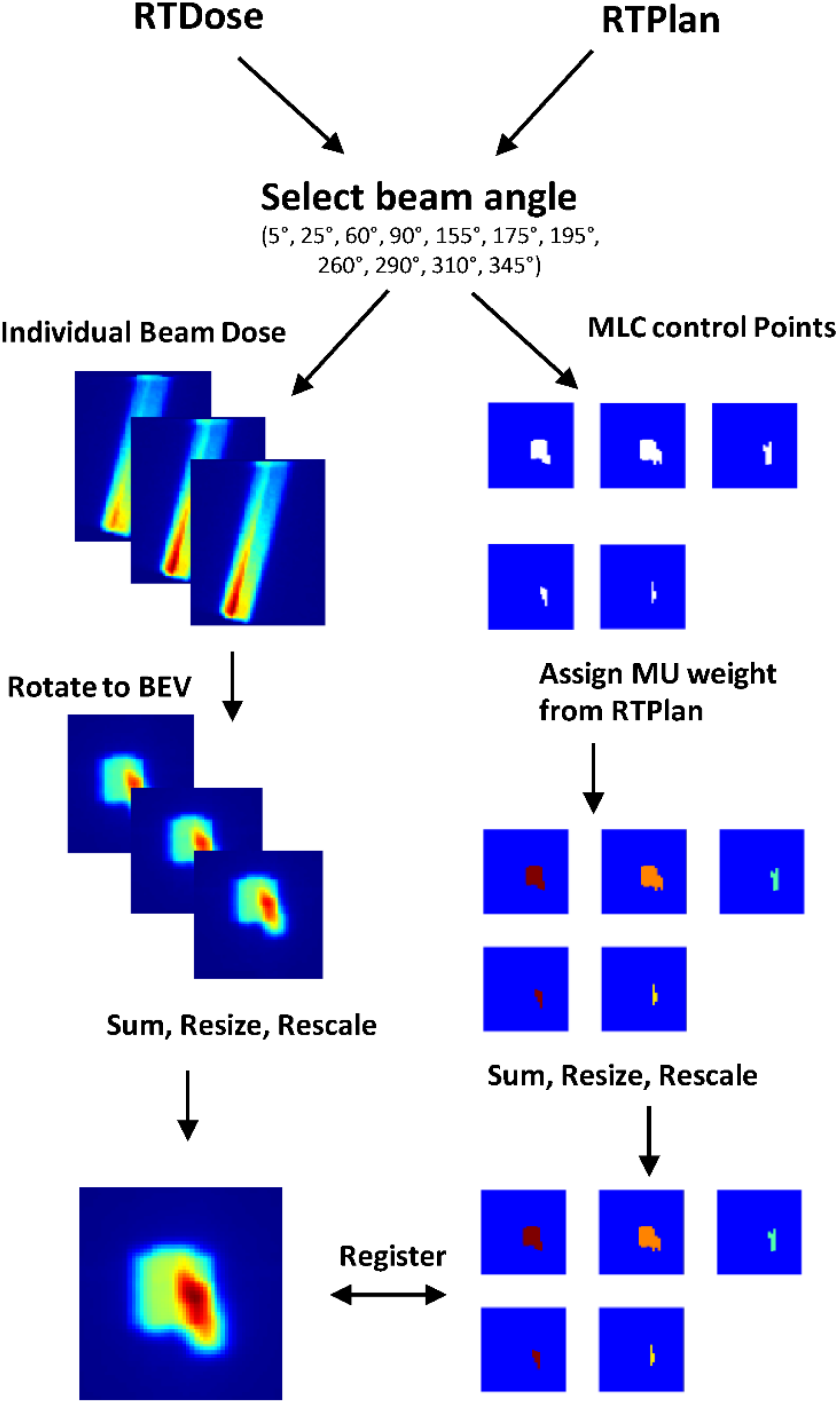
Pre-processing workflow of training data.

A summary of the pre-processing steps can be visualized in **Figure 3**. The ground truth 3D dose distribution from individual beams was extracted from the RTDose dicom and was resampled to the same dimensions and size as the daily planning MRI using MATLAB’s 3D interpolation. Then, the dose from each beam was shifted so that beam isocenter corresponds to the point of rotation for MATLAB’s rotation functions. The shifted dose distribution was then rotated towards the beam’s eye view (BEV), and summed along the BEV axis to form a 2D dose map. The 2D dose map was then resampled to [128,128,1] and rescaled between zero and one. Simultaneously, the MLC control points that define the corresponding field segment shape of each beam were extracted from the RTPlan dicom. For this collection of plans, the minimum number of segments per beam was one and the maximum segments per beam used was 12. The individual field segment shapes were reconstructed from MLC leaf positions as binary masks projected at isocenter where points blocked by the MLCs were zero and points in the open field were one using the matRad package for obtaining field shapes with 7.15 mm wide leaves. From the planning documents, the MU weight was multiplied by the binary matrix so that all points in the open field are the same MU value. These preprocessing steps resulted in registered 2D dose maps and MLC weighted apertures so that the DL network may learn the correct spatial relationship between dose and aperture shapes. Then each field segment was placed into a separate output channel. Since the number of ground truth field segments varied across each beam and patient, we fixed the number of output channels at six. If there were not enough segments to fill all channels, zero-padded matrices were used as a place holder. The final output of the model was resampled to [128,128,6]. All data preparation was completed using Matlab2019b (MathWorks).

### 2.3 Training, validation, and testing of deep learning models

The input and output of the generator were the 2D dose map and individual field segments, respectively. The input to the discriminator was the 2D dose map paired with either the ground truth field segments or the predicted field segments. While the generator penalized predicted field segments different from the ground truth, the discriminator was trained to classify NxN patches of the generator output as either synthetic or real data. This discriminator was trained on using 70x70 patch size was used as this was found to be a good compromise between computation time and performance. Since the model was conditioned on the 2D dose map from individual beams as opposed to the complete dose distribution from all 11 beams per plan, the training dataset size increased from 50 to 550. Patient-based leave-one-out-cross-validation was employed to test the robustness of the model, resulting in 10 separate models. For each model trained, four (44 training beams) out of five fractionated plans of the left-out patient were set aside for testing purposes. We deliberately kept a single fractionated plan in the training dataset so that the model could learn to replan the patient based on a prior plan. This validation strategy shows the feasibility of using our method to predict beam parameters for a new patient that is to be treated. To implement the method, an initial plan would be developed using conventional methods, and utilizing transfer learning, the model would be updated to include data from this conventionally produced plan. Future adaptive daily plans would be generated with the trained model using the daily image acquired and the corresponding dose distribution. The model was trained until stable; that is when the generator loss was approximately equal to the discriminator loss. Thus, we achieved stability using 200 epochs, a learning rate of 10^-3^, and a beta_1 rate of 0.5 using the TensorFlow Adam optimizer. Several techniques commonly known to promote GAN stability were implemented. For example, instead of using zeros to label synthetic data and ones to label real data, we used randomly generated “soft labels” with values between 0 and 1.2. We also made the labels noisy by randomly mislabeling synthetic and real data. In addition, we implemented spectral normalization in the discriminator, as this is known to improve the performance of the discriminator (Miyato, 2018). The GAN network was trained using the Adam optimizer with a learning rate of 0.0001. The loss function of the generator and discriminator was the mean absolute error and binary cross-entropy, respectively. Training and testing were performed using a GTX 1660TI GPU. After training, each model was tested on the remaining four fractions (44 training beams) of the respective test patient.

### 2.4 Plan generation

To convert field segment shape to MLC control points, the predicted field segments were overlayed on a 57.2 (linac X-axis) cm x 22 (linac Y-axis) cm grid representing the maximum field size of the Unity MR-Linac at isocenter. Beam shaping for the Unity allows leaf movement along the linac Y-axis and diaphragm movement along the linac X-axis. Thus, the X-axis of the predicted field segment was resampled to the projected leaf width at the machine isocenter. Custom code written in Matlab was used to detect the edges of the field segment shape along the X- and Y-axes and find the corresponding grid coordinates. A 2% intensity threshold was applied to the predicted field segment to reduce low-intensity noise. The MU weights for each field segment were assigned based on the maximum intensity of the predicted field segment. All points inside each open field segment were forced to the maximum intensity value. Finally, the RTPlan dicom file from the first fraction was overwritten with the new MLC control points and MU weights. The new RTplan dicom was loaded back into the Monaco TPS and the corresponding dose distribution was calculated on the daily MRI.

## 3 Results

**Figure 4:**
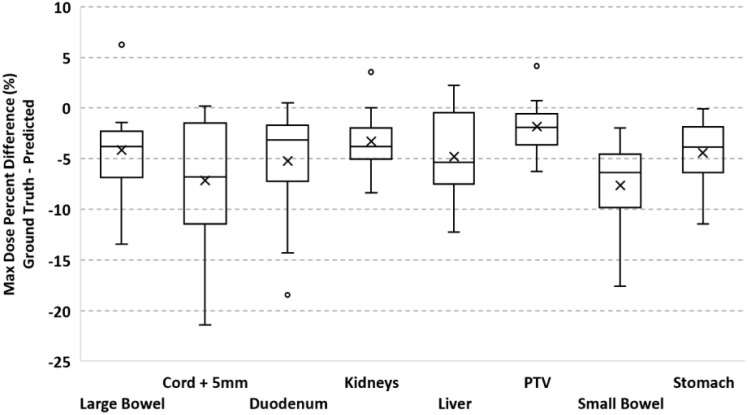
Boxplot of the max dose percent difference between the deep learning predicted plan and ground truth plan for the PTV and several OARs. For each test case, the max dose percent difference was averaged across FX2-FX5. Statistics for each ROI is representative of all test cases.

**Figure 5:**
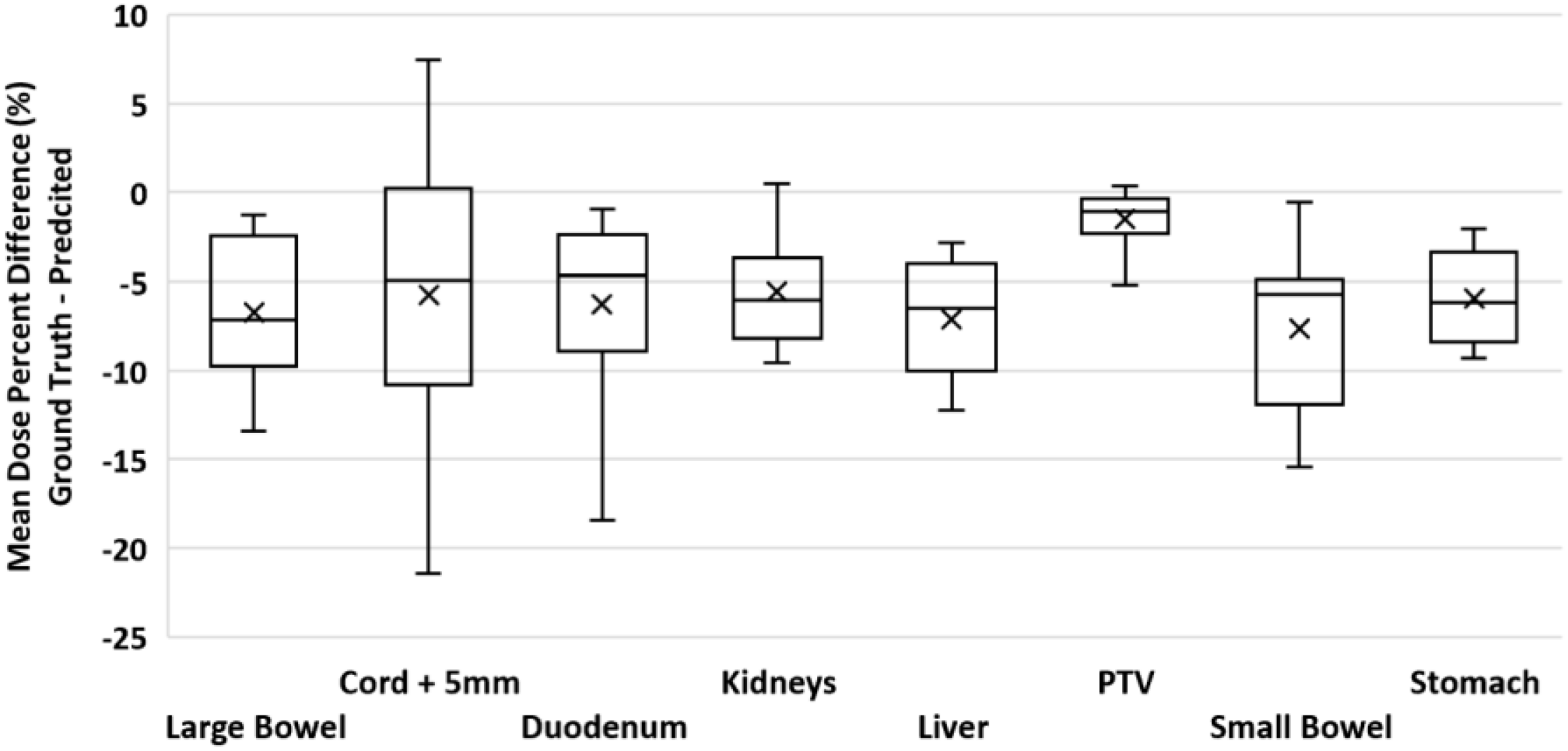
Boxplot of the mean dose percent difference between the deep learning predicted plan and ground truth plan for the PTV and several OARs. For each test case, the mean dose percent difference was averaged across FX2-FX5. Statistics for each ROI is representative of all test cases.

To validate our method, each of the ten models was evaluated on the remaining four fractions (44 training beams) of each patient’s treatment course. The dose distributions of the corresponding predicted plans were computed using the Monaco TPS. DVH analysis and gamma passing rates of the predicted dose distributions were compared to the ground truth plan’s dose distribution. Mean and max dose metrics were used to calculate the percent difference between the predicted plan and ground truth plan that was generated during MRgOART. The percent difference for the mean and max dose metrics and ROI were averaged across all four remaining test fractions of each patient. The distribution of the mean and max dose metrics across all patients is plotted in **Figures 4** and **5**. While the predicted plans maintain desired PTV coverage relative to the ground truth plans, the max and mean doses of the OARs are, on average, higher than the predicted plans by up to 7.6% (**Table 1**).

**Table 1:** Average max and mean dose percent difference (ground truth – predicted) of replans.

	Colon	Cord + 5mm	Duodenum	Kidneys	Liver	PTV	Small Bowel	Stomach
**Max Dose**	-4.2%	-7.1%	-5.2%	-3.3%	-4.8%	-1.9%	-7.6%	-4.4%
**Mean Dose**	-6.7%	-5.7%	-6.3%	-5.5%	-7.1%	-1.5%	-7.6%	-5.7%

The gamma passing rate (3%,3mm,5% threshold) was calculated between the predicted and ground truth plans for each fraction and test case (**Table 2**). Average gamma passing rates among all cases ranged from 80 to 95%.

**Table 2:** Gamma Analysis (3%,3mm,5% threshold): Percent passing rate of the predicted plan and ground truth plan.

Patient	1	2	3	4	5	6	7	8	9	10
FX2	88%	81%	100%	85%	87%	99%	82%	87%	88%	93%
FX3	93%	86%	89%	84%	92%	94%	89%	77%	95%	87%
FX4	89%	69%	95%	90%	99%	88%	–	78%	90%	89%
FX5	89%	84%	94%	95%	86%	84%	85%	97%	95%	81%
Average	90%	80%	95%	88%	91%	91%	85%	85%	92%	88%

**Figure 6:**
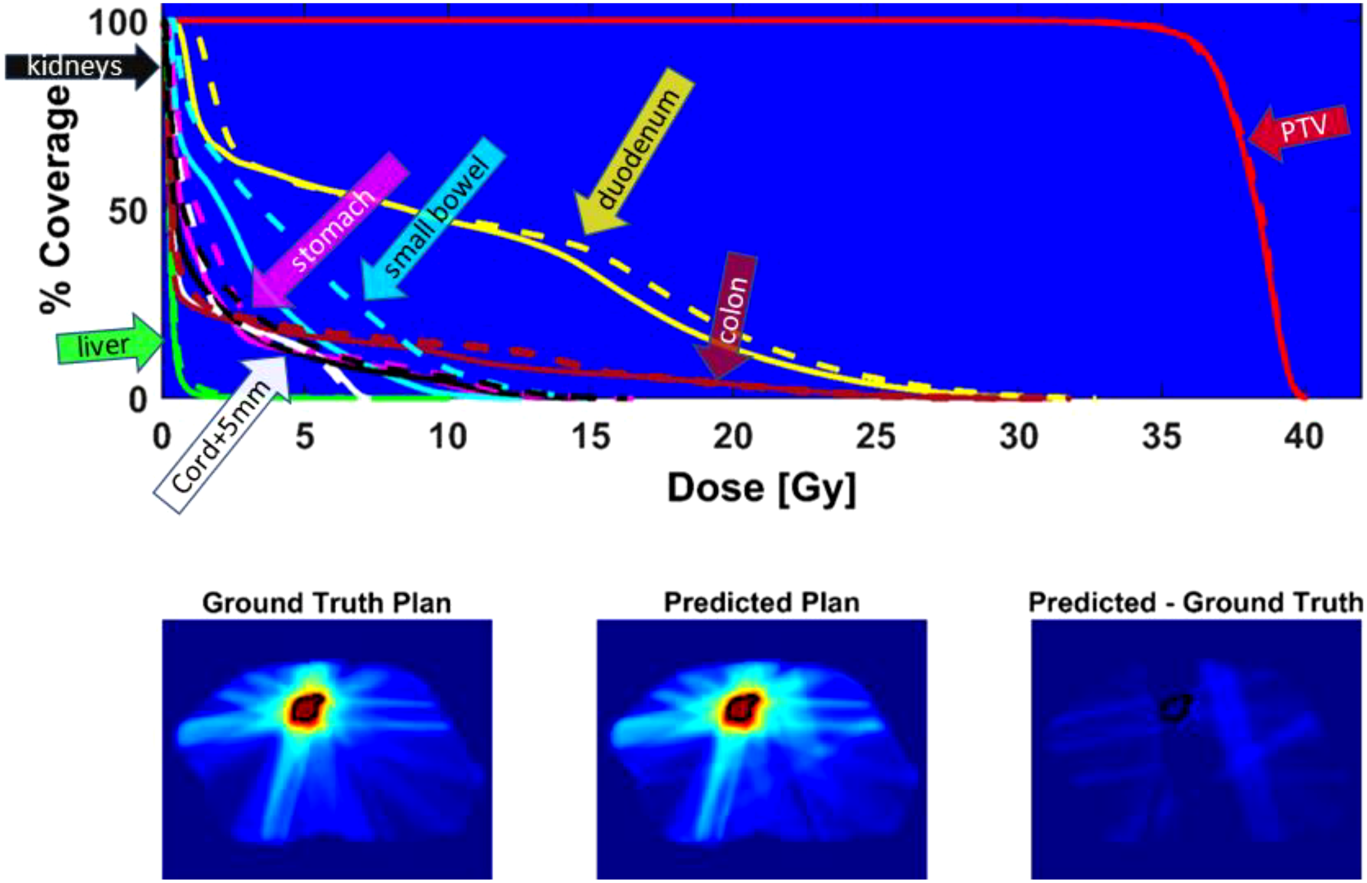
Top - DVH comparison for test case with average performance relative to Gamma passing rate: solid line = ground truth plan, dashed line = predicted plan. PTV (red), duodenum (yellow), colon (magenta), cord + 5mm (white), liver (green), small bowel (cyan), stomach (pink), kidneys (black). Bottom - center slice through ground truth dose distribution and predicted dose distribution and difference for a test case. The black contour is the PTV location.

**Figure 6** shows the DVH of the predicted plan and the ground truth plan of a better-performing test case. The corresponding center slices through each dose distribution and the subtraction is shown below, which demonstrates the similarity between predicted and ground truth plans.

The computation time required to predict field segment shapes and MU numbers for all 11 beams of each predicted plan was approximately 14 seconds (s). The conversion to a deliver RT dicom plan was an additional 6 s, for a total of approximately 20 s to automatically predict a deliverable plan.

**Figure 7:**
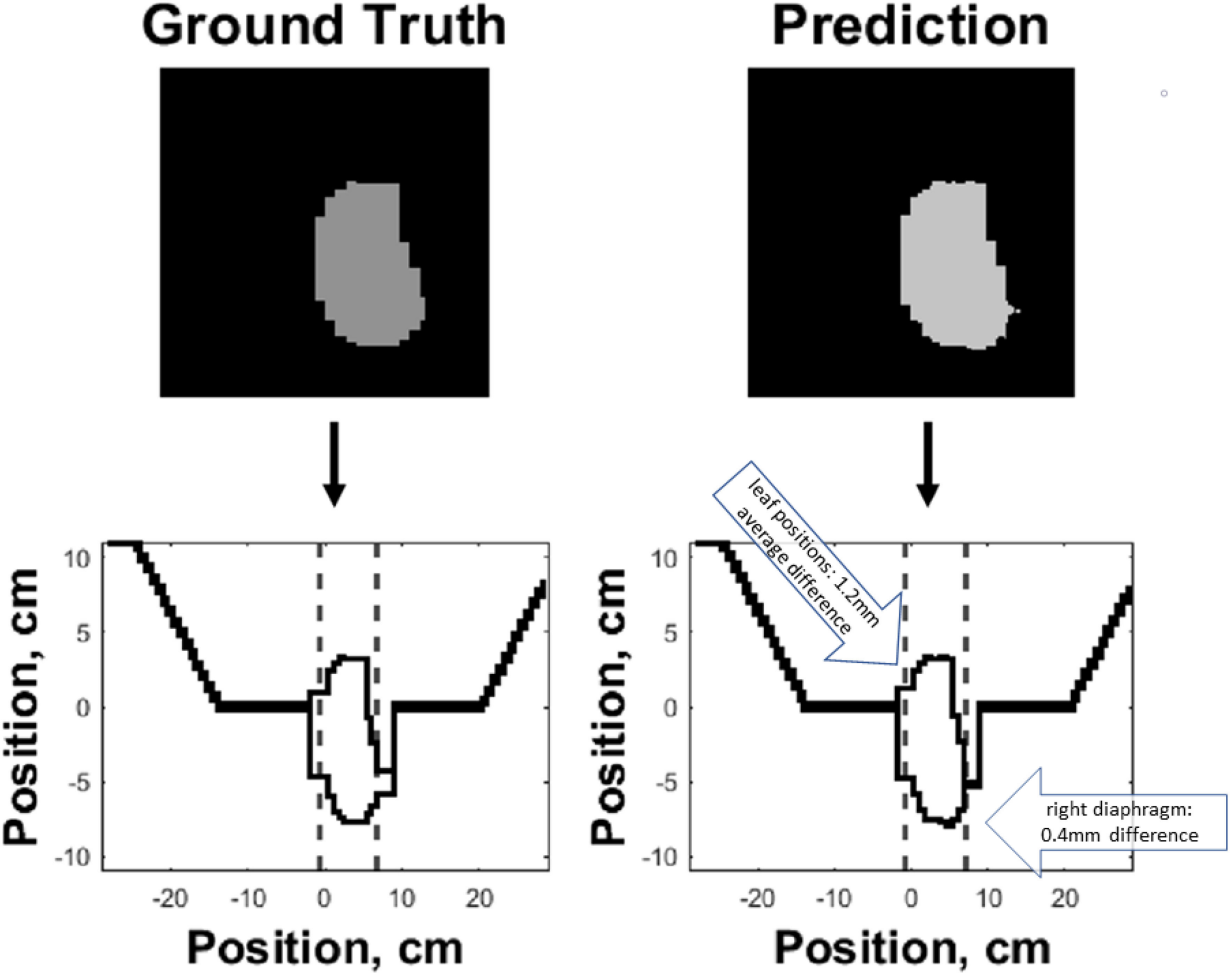
Side-by-side comparison of ground truth (left) and predicted (right) field segment shapes (top) and corresponding MLC control banks (bottom) of an example test case. The boundaries of the predicted and ground truth field segment shapes were used to define the corresponding MLC control points. The dashed lines along the horizontal axis of the two bottom plots represent the jaw positions, while the MLC leaf positions are represented along the vertical axis.

**Figure 7** shows an example of the predicted versus ground truth field segment shape and the corresponding MLC banks. As demonstrated in the figure, the outer boundaries of the field segment shape define the MLC control point locations. Custom Matlab (MathWorks) code was written to automatically detect the boundaries of the predicted field segment shapes and convert the shape to MLC control points.

**Figure 8:**
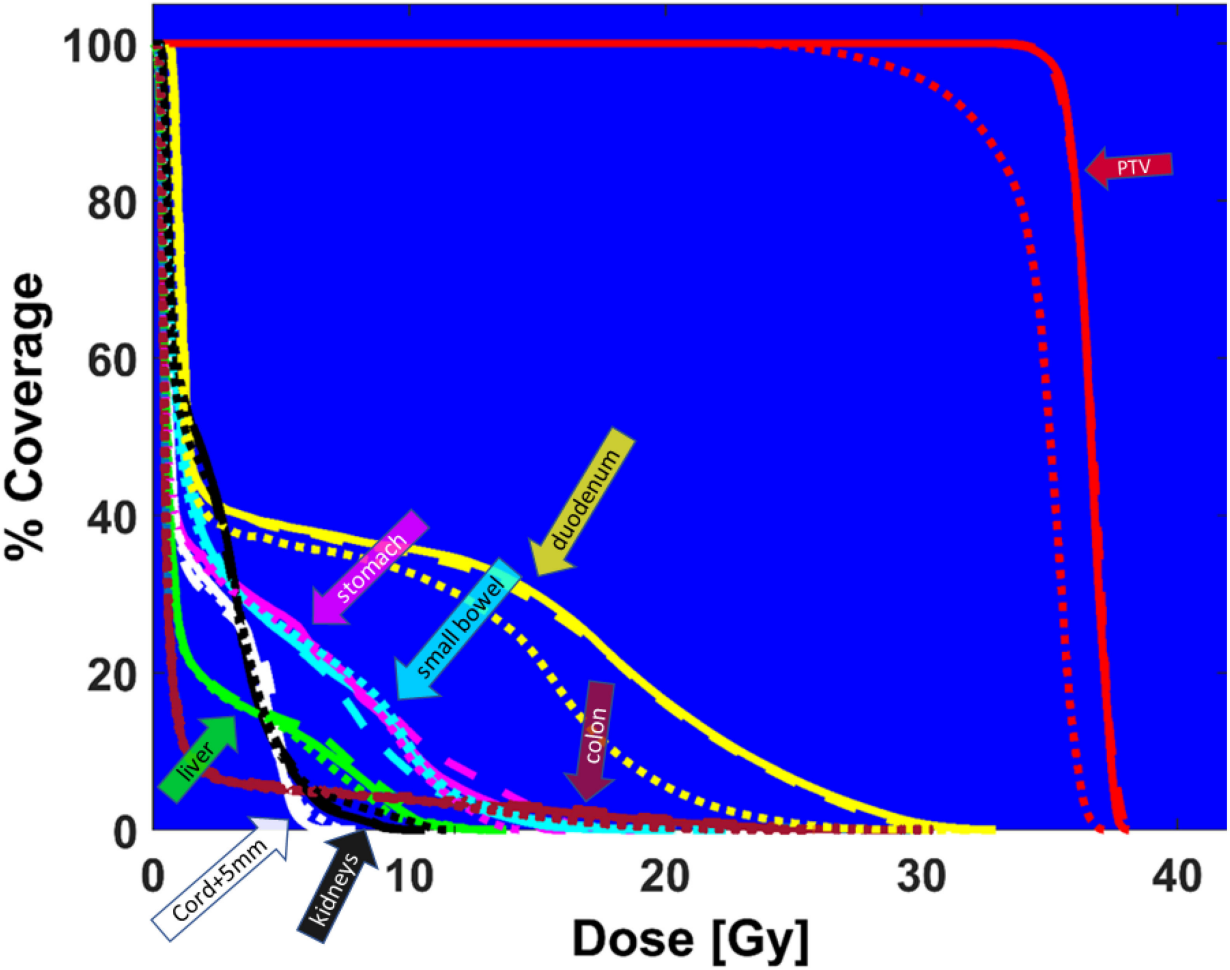
DVH comparison between ground truth plan (solid lines), predicted plan (large dashed lines), and reposition plan (small dashed lines) recalculated on daily MRI for PTV (red), duodenum (yellow), colon (magenta), cord + 5mm (white), liver (green), small bowel (cyan), stomach (pink), kidneys (black).

Finally, **Figure 8** demonstrates the high need for replanning to adapt to daily anatomy. Shown is the DVH of the predicted plan, the ground truth plan, and the reposition plan recalculated on daily MRI by shifting (repositioning) the reference plan based on the rigid-body registration of reference and the daily images. The predicted plan quality is comparable to the ground truth quality, but the DVH from the reposition plan does not have adequate PTV coverage. Although the predicted plan has slightly worse dose metrics compared to the ground truth plan, it is better than the reposition plan.

## 4 Discussion

This is an exploratory feasibility study investigating the potential of fast prediction of deliverable plan parameters based on DL, that may be potentially used for MRgOART or even RART. To the best of our knowledge, this is the first study that uses a cGAN to predict leaf sequencing, e.g., field segment shapes and MU numbers. Our work builds upon previous studies that use DL to predict desirable dose distributions based on patient anatomy (13–19). Given a new target dose distribution and reference plan, our newly proposed method can translate the target dose distribution to a deliverable plan with encouraging results. We believe that the agreement between the predicted and ground truth plans as well as the quality of the predicted plans can be improved with larger training datasets and incorporating the patient-specific anatomy (contours of lesion and OARs) as a part of model input. The greatest advantage of using DL to automatically predict deliverable adaptive plans is the little amount of time that is required. Each beam was sequenced in approximately 1 s. The computation time to predict treatment plans increases with the number of beams, but not plan complexity.

A key aspect of our method is that this is a replanning method, and not general planning from scratch. This allows the previously generated reference plan to be used as part of the training data, encouraging the network to learn from prior data. Also, because it is a replan, all beam angles of the newly predicted plan are unchanged from the reference plan. This eliminates the need to optimize beam angles, reducing the overall planning time.

In our feasibility study, we have elected to train our model using fractions that consistently have the same angles, with each fraction having 11 beams. Due to our current implementation being in the exploratory phase of our project, we found it reasonable to control for variabilities in angles and number of beams to isolate the impacts to deep learning of these variables. The plans we used to train our model are common in our clinic for treating Pancreatic cancer patients using SBRT. Future studies will utilize transfer learning to adapt our trained model to more patients, potentially having variable angles and number of beams. Our current goal is to show that our method is feasible as we adapt our models to more complicated set ups.

We have also chosen to train our model based on 2D cumulative projections of the 3D dose distribution as inputs. 3D dose distribution may be decomposed into 2D projections along relevant beam planes; thus our method may be practically implemented given a target dose distribution. There are currently vigorous efforts at predicting 3D dose distributions using patient anatomy (13–19), but these efforts have not been robustly translated to direct predictions of beam parameters for deliverable plans. Our current study is a step towards closing this gap, and future studies will incorporate patient anatomy directly.

With further development, the proposed method may be used for online replanning, and even for real-time intrafractional adaptation. In particular, we expect that as data from more patients is incorporated in the training of our deep learning models, the accuracy of our predictions will increase. We have observed this effect as we increased the limited number of patients we used for this study. Combining with on-gong efforts to speed up all other components of the replanning process, e.g., fast daily image acquisition and processing, robust auto-segmentation, automatic plan evaluation and verification, the present deep-learning adaptive plan generation method would substantially improve the efficiency of MRgOART.

## 5 Conclusion

Our findings indicate that it is feasible to use DL to predict deliverable adaptive plans quickly and automatically for online (or even real-time) adaptive replanning, without the need for a conventional lengthy inverse plan optimization process. Further studies using larger training dataset sizes and with the inclusion of patient-specific contours to the input of the network are needed to improve the accuracy and quality of the predicted adaptive plans.

## Data availability statement

The data analyzed in this study is subject to the following licenses/restrictions: Datasets will not be available. Requests to access these datasets should be directed to X. Allen Li, ali@mcw.edu.

## Ethics statement

The studies involving human participants were reviewed and approved by Institutional Review Board of Medical College of Wisconsin. Written informed consent for participation was not required for this study in accordance with the national legislation and the institutional requirements.

## Author contributions

XL and LB contributed to conception and design of the study. LB wrote the initial draft. SH managed revisions. LB, SH, YZ, XC contributed to data acquisition and analysis. All authors contributed to manuscript revision, read, and approved the submitted version.

## References

[1] Lagendijk JJ Raaymakers BW van Vulpen M. The magnetic resonance imaging–linac system. Semin Radiat Oncol (2014) 24:207–9. doi: 10.1016/j.semradonc.2014.02.009 24931095

[2] Ahunbay EE Peng C Chen GP Narayanan S Yu C Lawton C. An on-line replanning scheme for interfractional variations). Med Phys (2008) 35:3607–15. doi: 10.1118/1.2952443 18777921

[3] Paulson ES Ahunbay E Chen X Mickevicius NJ Chen GP Schultz C. 4D-MRI driven MR-guided online adaptive radiotherapy for abdominal stereotactic body radiation therapy on a high field MR-linac: Implementation and initial clinical experience. Clin Trans Radiat Oncol (2020) 23:72–9. doi: 10.1016/j.ctro.2020.05.002 PMC7256110 32490218

[4] Lamb J Cao M Kishan A Agazaryan N Thomas DH Shaverdian N. Online adaptive radiation therapy: Implementation of a new process of care. Cureus (2017) 9(8):e1618. doi: 10.7759/cureus.1618 29104835 PMC5663325

[5] Keiper TD Tai A Chen X Paulson E Lathuilière F Bériault S. Feasibility of real-time motion tracking using cine MRI during MR-guided radiation therapy for abdominal targets. Med Phys (2020) 47:3554–66. doi: 10.1002/mp.14230 32402111

[6] Batista V Richter D Chaudhri N Naumann P Herfarth K Jäkel O. Significance of intra-fractional motion for pancreatic patients treated with charged particles. Radiat Oncol (2018) 13:1060–8. doi: 10.1186/s13014-018-1060-8 PMC6020245 29941049

[7] Kumagai M Hara R Mori S Yanagi T Asakura H Kishimoto R. Impact of intrafractional bowel gas movement on carbon ion beam dose distribution in pancreatic radiotherapy. Int J Radiat OncologyBiologyPhysics (2009) 73:1276–81. doi: 10.1016/j.ijrobp.2008.10.055 19251100

[8] McIntosh C Purdie TG. Voxel-based dose prediction with multi-patient atlas selection for automated radiotherapy treatment planning. Phys Med Biol (2016) 62:415–31. doi: 10.1088/1361-6560/62/2/415 27997376

[9] McIntosh C Welch M McNiven A Jaffray DA Purdie TG. Fully automated treatment planning for head and neck radiotherapy using a voxel-based dose prediction and dose mimicking method. Phys Med Biol (2017) 62:5926–44. doi: 10.1088/1361-6560/aa71f8 28486217

[10] Cardenas CE Yang J Anderson BM Court LE Brock KB. Advances in auto-segmentation. Semin Radiat Oncol (2019) 29:185–97. doi: 10.1016/j.semradonc.2019.02.001 31027636

[11] Bibault JE Giraud P Burgun A. Big data and machine learning in radiation oncology: State of the art and future prospects. Cancer Lett (2016) 382:110–17. doi: 10.1016/j.canlet.2016.05.033 27241666

[12] Feng M Valdes G Dixit N Solberg TD. Machine learning in radiation oncology: Opportunities, requirements, and needs. Front Oncol (2018) 8:110. doi: 10.3389/fonc.2018.00110 29719815 PMC5913324

[13] Campbell WG Miften M Olsen L Stumpf P Schefter T Goodman KA. Neural network dose models for knowledge-based planning in pancreatic SBRT. Med Phys (2017) 44:6148–58. doi: 10.1002/mp.12621 PMC5734636 28994459

[14] Fan J Wang J Chen Z Hu C Zhang Z Hu W. Automatic treatment planning based on three-dimensional dose distribution predicted from deep learning technique. Med Phys (2018) 46:370–81. doi: 10.1002/mp.13271 30383300

[15] Kearney V Chan JW Haaf S Descovich M Solberg TD. DoseNet: a volumetric dose prediction algorithm using 3D fully-convolutional neural networks. Phys Med Biol (2018) 63:235022. doi: 10.1088/1361-6560/aaef74 30511663

[16] Nguyen D Long T Jia X Lu W Gu X Iqbal Z. A feasibility study for predicting optimal radiation therapy dose distributions of prostate cancer patients from patient anatomy using deep learning. Sci Rep (2019) 9:10. doi: 10.1038/s41598-018-37741-x 30705354 PMC6355802

[17] Barragán-Montero AM Nguyen D Lu W Lin MH Norouzi-Kandalan R Geets X. Three-dimensional dose prediction for lung IMRT patients with deep neural networks: robust learning from heterogeneous beam configurations. Med Phys (2019) 46:3679–91. doi: 10.1002/mp.13597 31102554

[18] Babier A Mahmood R McNiven AL Diamant A Chan TC. Knowledge-based automated planning with three-dimensional generative adversarial networks. Med Phys (2019) 47:297–306. doi: 10.1002/mp.13896 31675444

[19] Chen X Men K Li Y Yi J Dai J. A feasibility study on an automated method to generate patient-specific dose distributions for radiotherapy using deep learning. Med Phys (2019) 46(1):56–64. doi: 10.1002/mp.13262 30367492 PMC7379709

[20] Wang W Sheng Y Wang C Zhang J Li X Palta M. Fluence map prediction using deep learning models – direct plan generation for pancreas stereotactic body radiation therapy. Front Artif Intell (2020) 3:68. doi: 10.3389/frai.2020.00068 33733185 PMC7861344

[21] Lee H Kim H Kwak J Kim YS Lee SW Cho S. Fluence-map generation for prostate intensity-modulated radiotherapy planning using a deep-neural-network. Sci Rep (2019) 9:1–11. doi: 10.1038/s41598-019-52262-x 31666647 PMC6821767

[22] Ma L Chen M Gu X Lu W. Deep learning-based inverse mapping for fluence map prediction. Phys Med Biol (2020) 65:235035. doi: 10.1088/1361-6560/abc12c PMC8044255 33053515

[23] Guo Y Liu Y Georgiou T Lew MS. A review of semantic segmentation using deep neural networks. Int J Multimedia Inf Retrieval (2017) 7:87–93. doi: 10.1007/s13735-017-0141-z

[24] Isola P Zhu J Zhou T Efros A. Image-to-image translation with conditional adversarial networks. 2017 IEEE Conference on Computer Vision and Pattern Recognition (2017), 5967–76. doi: 10.1109/CVPR.2017.632

[25] Mahmood R Babier A McNiven A Diamant A Chan T. Automated treatment planning in radiation therapy using generative adversarial networks. Proceedings of the 3rd Machine Learning for Healthcare Conference (2018) 85:484–499.

[26] Ronneberger O Fischer P Brox T. U-Net: Convolutional networks for biomedical image segmentation. Medical Image Computing and Computer-Assisted Intervention (2015) 9351:234–41. doi: 10.1007/978-3-319-24574-4_28

